# Formyl-methionine as a degradation signal at the N-termini of bacterial proteins

**DOI:** 10.15698/mic2015.10.231

**Published:** 2015-09-06

**Authors:** Konstantin I. Piatkov, Tri T. M. Vu, Cheol-Sang Hwang, Alexander Varshavsky

**Affiliations:** 1 Division of Biology, California Institute of Technology, Pasadena, CA 91125, USA.; 2 Department of Life Sciences, Pohang University of Science and Technology, Pohang, Gyeongbuk, 790-784, South Korea.; 3 Center for Biotechnology and Biomedicine, Skolkovo Institute of Science and Technology, Moscow, 143026, Russia.

**Keywords:** bacteria, formyl-methionine, degron, N-end rule, cotranslational degradation

## Abstract

In bacteria, all nascent proteins bear the pretranslationally formed N-terminal formyl-methionine (fMet) residue. The fMet residue is cotranslationally deformylated by a ribosome-associated deformylase. The formylation of N-terminal Met in bacterial proteins is not strictly essential for either translation or cell viability. Moreover, protein synthesis by the cytosolic ribosomes of eukaryotes does not involve the formylation of N-terminal Met. What, then, is the main biological function of this metabolically costly, transient, and not strictly essential modification of N terminal Met, and why has Met formylation not been eliminated during bacterial evolution? One possibility is that the similarity of the formyl and acetyl groups, their identical locations in N terminally formylated (Nt formylated) and Nt-acetylated proteins, and the recently discovered proteolytic function of Nt-acetylation in eukaryotes might also signify a proteolytic role of Nt formylation in bacteria. We addressed this hypothesis about fMet based degradation signals, termed fMet/N-degrons, using specific *E. coli *mutants, pulse-chase degradation assays, and protein reporters whose deformylation was altered, through site-directed mutagenesis, to be either rapid or relatively slow. Our findings strongly suggest that the formylated N-terminal fMet can act as a degradation signal, largely a cotranslational one. One likely function of fMet/N-degrons is the control of protein quality. In bacteria, the rate of polypeptide chain elongation is nearly an order of magnitude higher than in eukaryotes. We suggest that the faster emergence of nascent proteins from bacterial ribosomes is one mechanistic and evolutionary reason for the pretranslational design of bacterial fMet/N degrons, in contrast to the cotranslational design of analogous Ac/N degrons in eukaryotes.

## INTRODUCTION

**Figure 1:**
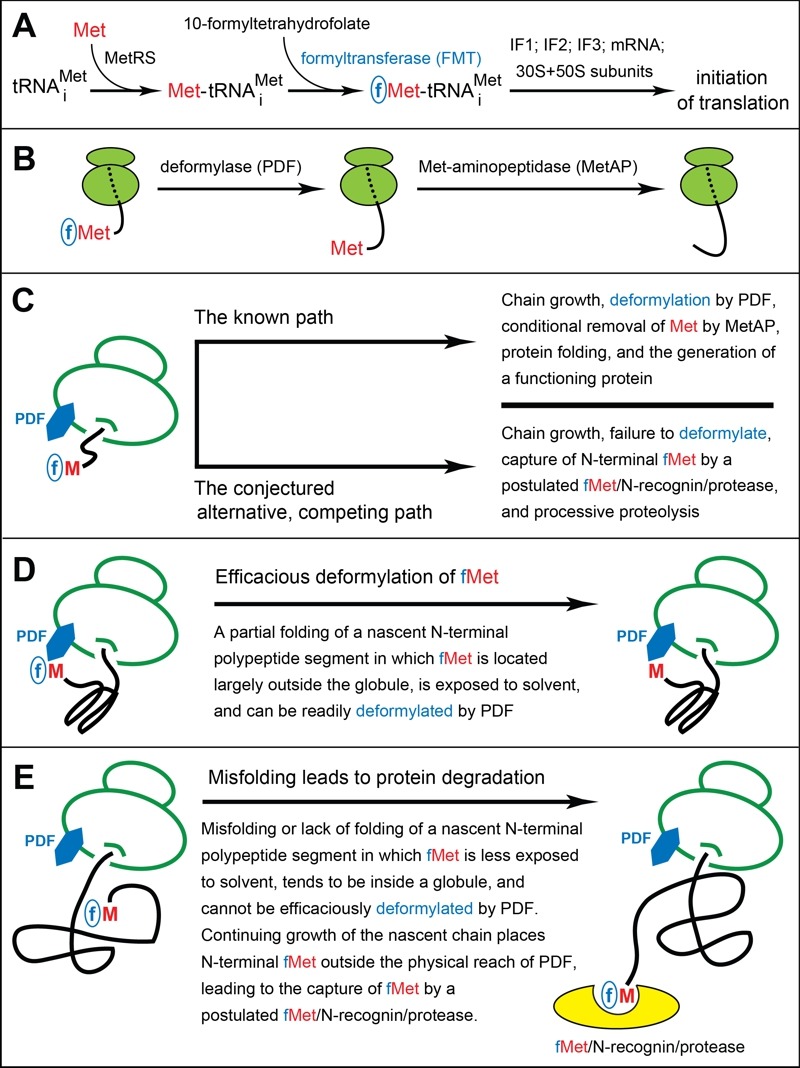
FIGURE 1: The working model of fMet/N-degrons. **(A)** Pretranslational enzymatic steps that result in formyl-Met (fMet) becoming the first residue of a nascent bacterial polypeptide. MetRS, Met-tRNA synthetase. IF proteins, initiation factors. **(B)** Translating ribosomes, with reversibly associated (not depicted) deformylase (PDF) and Met-aminopeptidase (MetAP) competing for their overlapping binding sites near the exit from the ribosomal tunnel. High affinity binding by the TF chaperone to a nascent polypeptide chain occurs once its length exceeds ~100 residues, usually after deformylation of N-terminal Met. A nascent chain, depicted unfolded in this diagram, tends to become unstably folded as it emerges from the tunnel. The rate of the MetAP-mediated removal of the deformylated N terminal Met residue depends on the identity of a residue at position 2. **(C-E)** Self-explanatory descriptions of the working model of fMet/N-degrons. See the main text for additional details and relevant citations.

Nascent polypeptides bear the N-terminal Met residue, encoded by the AUG initiation codon. In bacteria and in eukaryotic organelles mitochondria and chloroplasts (remote descendants of bacteria), this Met is N^α^-terminally formylated (Nt-formylated) through a “pretranslational” mechanism. Formyltransferase (FMT) uses 10 formyltetrahydrofolate to formylate the α-amino group of the Met moiety in the initiator tRNA_i_^Met^ 1,2,3,4,5,6,7,8. The resulting formyl-Met (fMet) becomes the first residue of a nascent polypeptide that emerges from a bacterial ribosome (Fig. 1A) 9,10,11,12,13. The formyl moiety of N-terminal fMet is cotranslationally removed by peptide deformylase (PDF), which is reversibly bound to the ribosome near the exit from the ribosomal tunnel (Fig. 1B) 4,14,15,16,17,18,19,20,21,22,23,24,25,26,27. A ribosome-associated chaperone called trigger factor (TF) interacts with proteins emerging from the tunnel 28,29,30,31,32,33,34,35,36,37,38,39,40,41,42. The signal recognition particle (SRP) also binds to some nascent proteins, recognizing specific sequence motifs (signal sequences) and directing SRP-associated proteins for translocation through the inner membrane 42,43,44,45.

Once N terminal fMet of a nascent protein is deformylated by PDF, the resulting Met can be cleaved off by Met aminopeptidase (MetAP) (Fig. 1B). The removal of (deformylated) Met by MetAP requires that a residue at position 2, to be made N terminal by the cleavage, is not larger than Val 26,46,47,48. The *Escherichia coli* PDF binds to the 50S ribosomal subunit in part through contacts with the L22 ribosomal protein 23,26. PDF and MetAP act sequentially in their cotranslational processing of nascent proteins and compete with each other for interactions with their overlapping binding sites on the ribosome near the tunnel’s exit 26.

High-affinity interactions of the TF chaperone with a nascent protein begin to take place after the first ~100 residues of the protein have been synthesized 28. Deformylation of N terminal fMet by PDF (Fig. 1B) is impeded in cells engineered to overproduce TF 28. Consequently, it is likely that in wild-type cells, by the time a nascent protein becomes larger than ~100 residues, i.e., shortly before the binding of TF to this protein 28, its N terminal fMet had already been, in most cases, deformylated by the ribosome associated PDF. The rate of chain elongation by bacterial ribosomes *in vivo* at 37°C is 10 20 residues/sec 49,50,51,52. Thus, the *in vivo* lifespan of the formyl group, from the moment fMet becomes the first residue of a newly initiated protein to the moment of fMet deformylation, is usually less than a minute. Given the delay in high affinity binding of TF to a nascent protein 28, its first ~100 residues, which require 5-10 sec to be made, may be unassociated, during a fraction of those 5 - 10 sec, with any chaperone.

Although the fMet moiety of bacterial fMet tRNA_i_^Met^ interacts with the initiation factor IF2 and thereby contributes to the efficacy of translation initiation 5,7,53,54,55, the formylation of N terminal Met is not strictly essential for protein synthesis and cell viability. For example, *E.coli* *fmt* mutants lacking formyltransferase are viable. Their abnormal phenotypes include slow growth and hypersensitivity to stresses 3,4,7. In *Salmonella enterica*, the slow growth of *fmt* mutants can be alleviated, during serial passaging, through the emergence of mutants that overexpress the initiator tRNA_i_^Met^ 56. In *Pseudomonas aeruginosa*, an engineered overexpression of IF2 can alleviate slow growth of *fmt* mutants in minimal media 57. Moreover, in *P. aeruginosa* and some other bacteria (other than *E. coli*), the ablation of *fmt* results in cells whose growth rates in rich media are nearly identical to those of wild-type cells 57,58.

In contrast, deformylation of the bulk of N terminal fMet in nascent proteins is required for cell viability. Either a strong inhibition of PDF by the antibiotic actinonin or ablation of the PDF encoding *def* gene are lethal, because MetAP is unable to cleave off the formyl-bearing N terminal fMet 46. (The inability to remove N-terminal Met leads to cell death in part because specific non Met residues, e.g., Thr, must become N-terminal in some essential enzymes, in which these non-Met residues are parts of enzymes’ active sites 59.) However, double *fmt def* mutants, which lack both FMT and PDF and therefore can neither deformylate fMet nor formylate it in the first place, are viable, with phenotypes similar to those of single *fmt* mutants 23.

In eukaryotes, protein synthesis by the cytosolic ribosomes does not involve the formylation of Met, indicating that it was feasible, during evolution, to either lose the formylation of Met or not to acquire it in the first place. (It is unknown whether formylation of Met was a part of translation in the last common ancestor of extant organisms.) Innate immune responses involve the recognition of Nt-formylated bacterial proteins and short peptides. They are present in infected animals at high enough levels to act as chemoattractants for macrophages and neutrophils 60,61. Consequently, the formylation of Met can be a detriment to bacterial fitness.

Given these properties of fMet, why do all examined wild-type bacteria contain formyltransferase, deformylase, and use fMet, rather than Met, to initiate translation? Why has this pervasive, metabolically costly, transient, and not strictly essential modification of N terminal Met not been eliminated during bacterial evolution? This conundrum suggested to us that the main biological function of fMet, the one that underlies the universal presence of N terminal fMet in extant wild type bacteria, remained to be discovered.

Previous work identified the N-terminus of an intracellular protein as the site of degradation signals (degrons 62) that are targeted by the N-end rule pathway (Fig. S1). This pathway is a set of proteolytic systems whose unifying feature is their ability to recognize proteins containing N terminal degradation signals called N-degrons and to cause the processive degradation of such proteins by the 26S proteasome in eukaryotes (Fig. S1A, B) 63,64,65,66,67,68,69,70,71,72,73,74 or by the proteasome-like protease ClpAP in bacteria (Fig. S1C, D) 75,76,77,78,79,80. In eukaryotes, N-degrons can also mediate the degradation of specific proteins (and their noncovalently bound protein ligands) by autophagy, as distinguished from the proteasome 74. The main determinant of an N-degron is either an unmodified or chemically modified “destabilizing” N terminal residue of a protein. Recognition components of the N-end rule pathway are called N recognins. In eukaryotes, N recognins are specific E3 ubiquitin (Ub) ligases that recognize N degrons and polyubiquitylate proteins bearing them 71,72,73. Bacteria lack the bona fide Ub system. The bacterial N-end rule pathway employs the ClpS N-recognin (but no ubiquitylation) to deliver targeted N-end rule substrates to the ClpAP protease (Fig. S1C, D) 75,76,79,80,81,82,83,84,85,86,87,88,89,90,91,92,93.

In eukaryotes, the N-end rule pathway consists of two branches. One of these branches, called the Arg/N-end rule pathway, targets proteins bearing N terminal Arg, Lys, His, Leu, Phe, Tyr, Trp, Ile, Asn, Gln, Asp, Glu, and Cys (Fig. 1B) 63,65,71,72,73,94,95,96,97. This pathway can also target unmodified N-terminal Met, if Met is followed by a bulky hydrophobic residue (Fig.S1A). Among these N-terminal residues, Asn, Gln, Asp, Glu, and Cys are destabilizing owing to their preliminary enzymatic modifications, which include N terminal deamidation (Nt deamidation) of Asn and Gln and Nt-arginylation of Asp, Glu and Cys (the latter after its conditional oxidation) 66,96,98,99. The substrate specificity of the bacterial N-end rule pathway is similar to the targeting range of the Arg/N-end rule pathway but is not as broad (Fig.S1C, D) 71,77,78.

The other branch of the eukaryotic N end rule pathway is called the Ac/N-end rule pathway. It recognizes proteins through their N^α^ terminally acetylated (Nt-acetylated) residues (Fig. S1B) 67,68,69,70. The degrons and N-recognins of the Ac/N-end rule pathway are called Ac/N degrons and Ac/N recognins, respectively. Nt-acetylation of eukaryotic proteins is largely cotranslational, being mediated by ribosome-associated Nt-acetylases 100,101,102. At least 60% and about 90% of proteins are Nt-acetylated in the yeast *S. cerevisiae* and in human cells, respectively 103,104,105,106. Nt-acetylation is apparently irreversible, i.e., a protein molecule acquires the N^α^-acetyl group largely at birth and retains this group for the rest of that molecule’s lifetime in a cell. While Nt-acetylation also takes place in bacteria, it involves less than 10% of bacterial proteins and can occur only after the PDF-mediated deformylation of N-terminal fMet 107,108. Nothing is known about whether or not a version of the Ac/N-end rule pathway exists in bacteria as well.

The acetyl and formyl groups differ by the CH_3_ moiety vs. the hydrogen atom. It occurred to us that the similarity of acetyl and formyl, their identical locations in Nt acetylated and Nt-formylated proteins, and the recently discovered proteolytic function of Nt acetylation in eukaryotes 67,68,69,70,71 might also signify the proteolytic role of Nt formylation in bacteria, despite the transiency of the formyl group in fMet of nascent bacterial proteins. We proposed this hypothesis in 2010 67 and carried out experiments to verify it in the present study.

**Figure 2:**
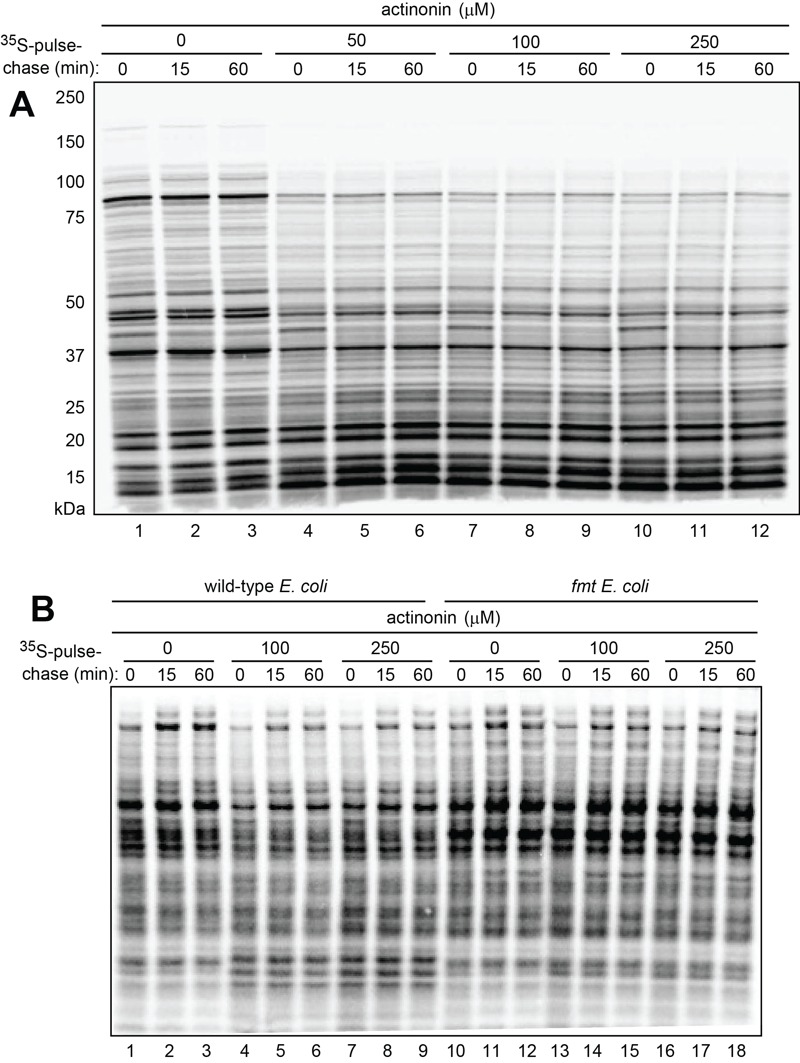
FIGURE 2: Pulse-chase analyses in wild-type and formylation-lacking *fmt* *E. coli* in the absence or presence of actinonin, an inhibitor of deformylation. **(A)** Wild type *E. coli* were pulse-labeled with ^35^S-methionine/cysteine for 1 min, followed by a chase (in the presence of chloramphenicol, a translation inhibitor) for indicated times, extraction of proteins, SDS-PAGE, and autoradiography. Pulse-chases were carried out either in the absence of actinonin (lanes 1-3) or in the presence of increasing concentrations of actinonin (lanes 4-12). Molecular masses of protein markers are indicated on the left. **(B)** Same as in (A) but pulse chases were carried out in the absence of chloramphenicol in wild-type cells (lanes 1-9) and congenic *fmt* *E.coli* (lanes 10-18).

**Figure 3:**
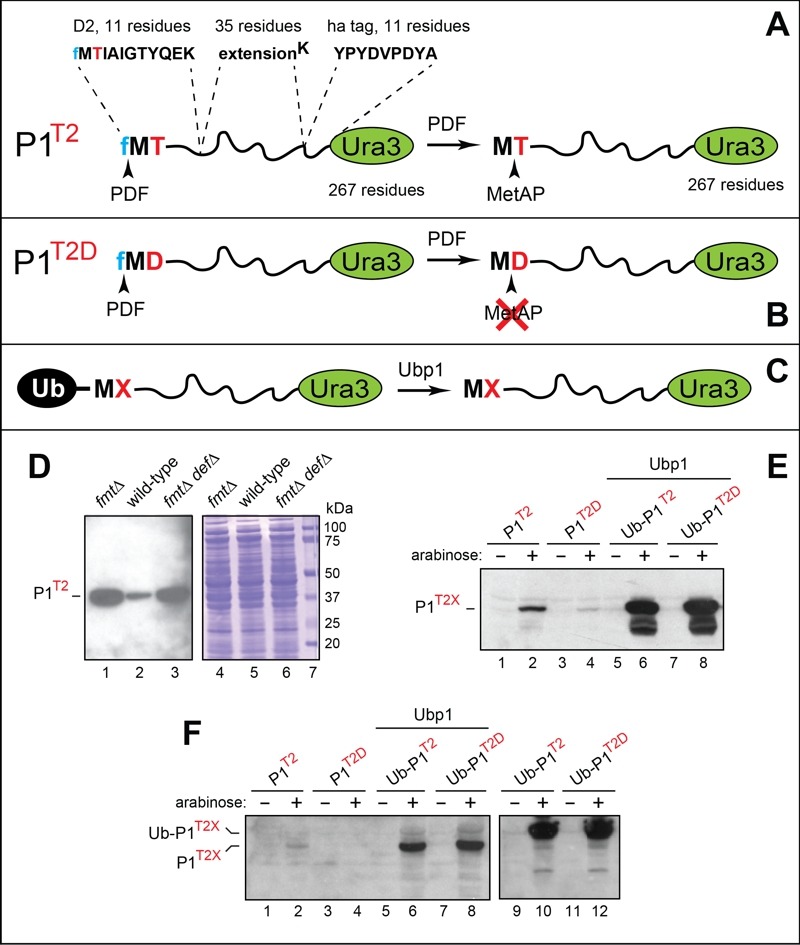
FIGURE 3: Analyses of reporter proteins in wild-type and formylation-lacking *fmt* *E.coli*. **(A)** Design of the P1^T2^ (D2^1-11^-eK-ha-Ura3) reporter protein. The term P1^T2^ (protein-1 containing Thr at position 2) denotes a chimeric reporter containing the indicated N terminal segments upstream of the 267-residue *S. cerevisiae* Ura3 moiety. Arrowheads indicate deformylation of N-terminal fMet by the PDF deformylase and the subsequent removal of Met by MetAP. See the main text for details. **(B)** Same as in (A) but the otherwise identical P1^T2D^ reporter contains Asp (D) at position 2. The rate of PDF-mediated deformylation of N-terminal fMet with Asp at position 2 is at least 10-fold lower than the rate of deformylation with Thr at position 2. Another difference between P1^T2^ and P1^T2D^ is the retention of N-terminal Met in P1^T2D^. See the main text for details and citations. **(C)** The use of ubiquitin (Ub) fusions to generate P1^T2^ and P1^T2D^ through the removal of the N-terminal Ub moiety by the *S. cerevisiae* Ubp1 deubiquitylase expressed in *E. coli*. **(D)** Immunoblotting analyses, after SDS-PAGE, of the P1^T2^ reporter protein expressed in *fmt* (lane 1), wild-type (lane 2) and *fmt def* (lane 3) *E. coli*. Lanes 4-6, the corresponding total protein patterns (Coomassie staining). Lane 7, molecular mass markers. **(E)** Immunoblotting analyses, after SDS-PAGE, of the P1^T2^ and P1^T2D^ reporters expressed from the P*_ara_* promoter in wild-type *E. coli* (lanes 1-4), and of the Ub fusions Ub-P1^T2^ and Ub-P1^T2D^ in wild-type *E. coli* expressing the *S. cerevisiae* Ubp1 deubiquitylase (lanes 5-8). **(F)** Same as in (E), but independent experiments, in addition to expressing Ub-P1^T2^ and Ub-P1^T2D^ in the absence of coexpressed yeast Ubp1 (lanes 9-12). See the main text for details and citations.

**Figure 4:**
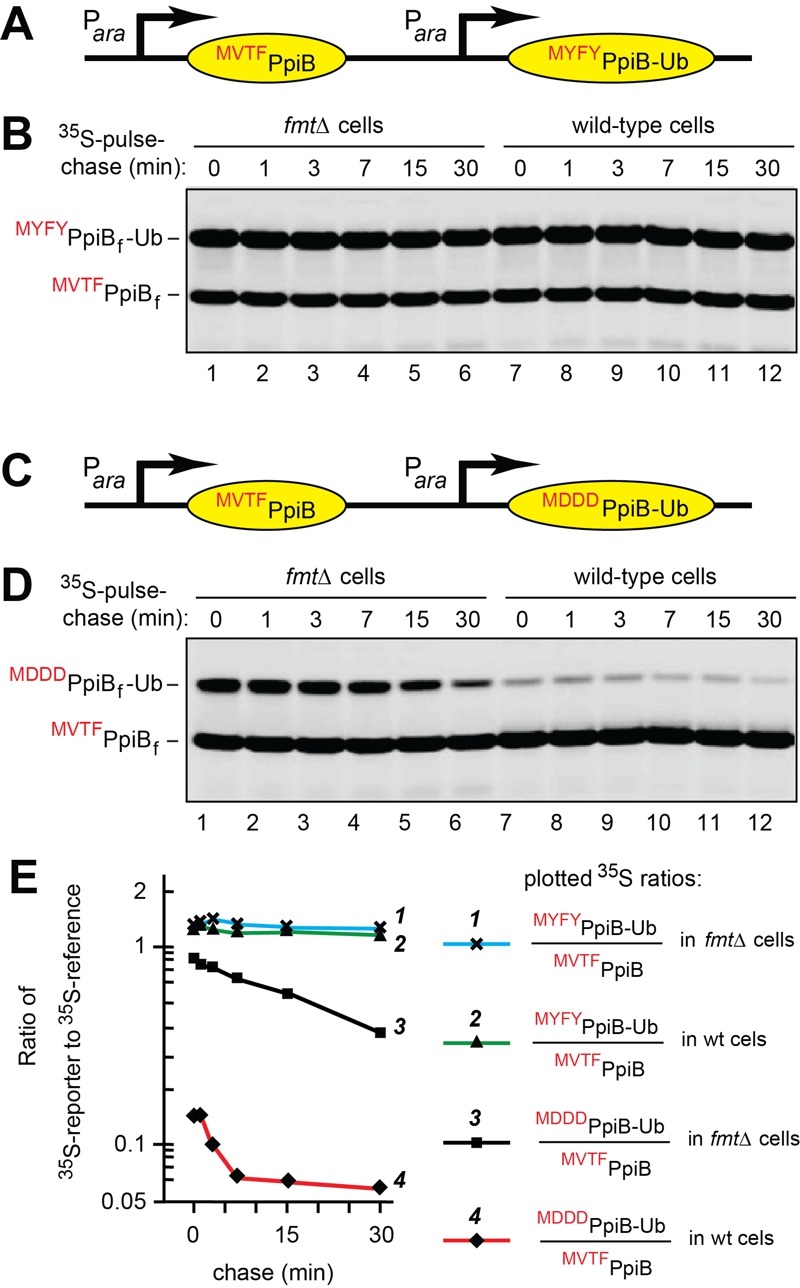
FIGURE 4: Formylation-dependent selective destabilization of PpiB-based reporter proteins. **(A)** Diagram of the expression cassette in which two identical tandem P*_ara_* promoters express the C-terminally flag-tagged PpiB reference protein ^MVTF^_wt_PpiB_f_ and the reporter protein ^MYFY^PpiB_f_ Ub (reporter-1). The latter differs from ^MVTF^_wt_PpiB_f_ by three amino acid residues adjacent to N terminal fMet, and by the presence of C-terminal Ub moiety, added to make the two proteins distinguishable by size. **(B)** Lanes 1-6, formylation-lacking *fmt* *E. coli* were pulse-labeled with ^35^S-methionine/cysteine for 1 min, followed by a chase (in the absence of chloramphenicol) for indicated times, extraction of proteins, immunoprecipitation with a monoclonal anti-flag antibody, SDS-PAGE, and autoradiography. Lanes 7-12, same but in wild type *E. coli*. **(C)** Same as in (A), with the reference ^MVTF^_wt_PpiB_f_ and the reporter ^MDDD^PpiB_f_ Ub (reporter-2), which differed from ^MYFY^PpiB_f_ Ub in (A) and (B) by three residues (Asp Asp-Asp) downstream from N-terminal fMet. **(D)** Same as in (B) but with ^MVTF^_wt_PpiB_f _ and ^MDDD^PpiB_f_ Ub (reporter-2). See the main text for the logic and details of these experiments.

The evidence below (Figs. 2-4) strongly suggests that N-terminal fMet can act as an N degron, termed fMet/N-degron. In bacteria, the rate of polypeptide chain elongation is nearly an order of magnitude higher than in eukaryotes. We suggest that the faster emergence of nascent proteins from bacterial ribosomes may be the mechanistic and evolutionary reason for the pretranslational design of bacterial fMet/N-degrons (Fig. 1A), in contrast to the cotranslational design of Ac/N-degrons in eukaryotes (Fig. S1B). By analogy with Ac/N-degrons 67,68,69,70,71, one function of bacterial fMet/N degrons is likely to be the quality control of both nascent proteins and just released, newly formed proteins. Specifically, fMet/N-degrons are envisioned to augment the quality of bacterial proteome through a preferential and largely cotranslational degradation of Nt-formylated misfolded proteins. This would happen at the price of eliminating a subset of normal proteins as well, given the stochasticity of both the PDF-mediated deformylation of fMet and the alternative, competing process of targeting and destroying Nt-formylated proteins through their fMet/N-degrons.

## RESULTS

### Inhibition of fMet deformylation decreases the levels of larger pulse-labeled proteins

Wild-type *E. coli* were pulse-labeled for 1 min at 37°C with ^35^S-methionine/cysteine in Fig. 2A. The pulse was followed by a chase (in the presence of translation inhibitor chloramphenicol), preparation of cell extracts, SDS PAGE, and autoradiography. Actinonin, a specific inhibitor of PDF, was either absent or present, at indicated concentrations, throughout pulse-chases. The inhibition of fMet deformylation by actinonin was found to cause a significant decrease in the levels of larger (more than ~35 kDa)^ 35^S labeled proteins and a concomitant increase of smaller (less than ~20 kDa) proteins (Fig. 2A).

Additional ^35^S-pulse-chases (this time in the absence of chloramphenicol) with wild-type vs. formyltransferase-lacking *fmt* *E. coli *showed that the above effect of actinonin required the formylation of N-terminal Met, because ^35^S-protein patterns in *fmt* cells were essentially the same in the presence or absence of actinonin (Fig. 2B). These results (Fig. 2) were consistent with the fMet/N degron hypothesis (Fig. 1C-E), as it predicts that the probability of destruction of an fMet-bearing nascent protein would be higher, on average, for a larger protein, because its polypeptide chain, i.e., its ribosome-associated peptidyl tRNA, would dwell in the vicinity of a translating ribosome for a longer time than would be the case for a smaller fMet bearing nascent protein. The postulated fMet/N-recognin/protease (Fig. 1E) or at least its fMet/N-recognin part is envisioned to be reversibly associated with the ribosomes (see Discussion). Thus, the probability of capture of larger Nt-formylated proteins by this protease would be higher than the corresponding probability for smaller fMet-bearing nascent proteins, because the latter would be released sooner and diffuse into the bulk solvent, i.e., into regions with (presumably) lower levels of the fMet/N recognin/protease.

An alternative interpretation of these results is that actinonin might increase the probability of premature chain termination. This increase would lead to a lower relative abundance of larger (as compared to smaller) pulse-labeled proteins in the presence of actinonin, thereby accounting for our results (Fig. 2) without invoking a preferential degradation of these proteins. However, this interpretation was made unlikely by the fact that the observed effect of actinonin required the formylated state of N terminal Met, i.e. this effect of actinonin was not observed with *fmt* cells, in contrast to wild-type cells (Fig. 2B).

### Higher levels of a protein reporter in formylation-lacking mutants

One prediction of the fMet/N-degron hypothesis is as follows: even in the case of a nascent protein whose N terminal amino acid sequence makes it an efficacious PDF substrate, some molecules of this protein would still be expected to be destroyed through the protein’s fMet/N-degron, given the stochasticity of deformylation of N-terminal fMet by PDF and the alternative, competing process of targeting an fMet-bearing protein for degradation (Fig. 1C-E). Consequently, the ribosome-associated PDF deformylase would be expected to occasionally lose the competition for N-terminal fMet to the postulated fMet/N-recognin/protease, resulting in the degradation of a targeted nascent protein. The kinetic advantage of PDF may be decreased if a nascent N-terminal segment of a protein would be either unfolded (with N-terminal fMet partly obscured within a “molten globule”) or misfolded in a way that decreases the time-averaged solvent exposure of N terminal fMet (Fig. 1C-E and Discussion). If so, the steady-state level of such a protein would be expected to increase in formyltransferase-lacking *fmt* mutants.

We addressed this prediction through a reporter bearing the N-terminal sequence of a protein called D2. Earlier studies of D2 protein in plant chloroplasts, by Giglione, Meinnel and colleagues 109,110, were relevant to experiments of the present study. Although our 2010 hypothesis about fMet as a degradation signal 67 was cited by Giglione and colleagues 110, they did not interpret their findings with D2 protein in terms of fMet/N-degrons. In contrast, the results below, using the N-terminal segment of D2 as a part of protein reporters (Figs. 3 and 4), strongly suggest that the data by Giglione *et al.* 109,110 can be interpreted, in hindsight, as a likely example of protein degradation mediated by fMet/N-degrons.

Our 37-kDa reporter, termed P1^T2^ (“protein-1 containing Thr at position 2”), comprised the 11-residue N terminal sequence MTIAIGTYQEK of the wild-type D2 protein (D2^1-11^), followed by the sequence GSGAWLLPVSLVKRKTTLAPNTQTASPRALADSLMQLARQVSRG (a 45-residue segment derived from the previously used e^K^ sequence [extension (e) containing lysine (K)] 67,71,111), by the 9-residue ha epitope tag (YPYDVPDYA), by the AFLGQ linker 67, and by the 267 residue Ura3 protein of the yeast *Saccharomyces cerevisiae* (Fig. 3A). The Ura3 moiety is a frequently employed component of protein reporters 67,69. The e^K^ segment is another sequence often used in reporters, in part because e^K^ is conformationally disordered while lacking degrons in both *E. coli* and *S. cerevisiae* 67,71,111.

P1^T2^ was expressed from the constitutive P*_KmR_* promoter in wild-type, null *fmt*, and null *fmt def* *E.coli* strains, followed by extraction of proteins, SDS-PAGE, and immunoblotting with anti-ha antibody (Fig. 3A, D). Extracts were adjusted for equal total loads using Bradford assay 112 and Coomassie staining of proteins fractionated by SDS-PAGE (Fig. 3D). The levels of the P1^T2^ reporter in both *fmt* and *fmt def* cells were strikingly higher than in congenic wild-type cells, in agreement with the above prediction of the fMet/N degron hypothesis (Fig. 3A, D).

The ~70-residue N-terminal segment (D2^1 11^ e^K^ ha) of the 37 kDa P1^T2^ reporter is a biologically irrelevant mix of different sequences (Fig. 3A). A nascent protein exemplified by P1^T2^, with its disordered N-terminal region, may be less amenable to the PDF mediated deformylation and would be, therefore, a relatively favored target for the capture and degradation by the postulated fMet/N-recognin/protease in fMet-containing wild-type cells (see Discussion for a more detailed exposition). Conversely, one would expect that an up regulation of such a reporter in formyltransferase-lacking cells may be particularly high, in agreement with the observed increase of P1^T2^ in *fmt* and *fmt def* *E. coli* (Fig. 3A, D).

### Bypass of Met formylation can equalize the levels of efficacious and poor substrates of deformylase.

When the D2 protein, encoded by chloroplast DNA, is expressed in chloroplasts, it bears the formylated N-terminal fMet, similarly to nascent bacterial proteins. The fMet of D2 is deformylated by two functionally overlapping PDFs in chloroplasts 21. The Thr residue at position 2 of the D2 protein (denoted as D2^T2^) becomes its N terminal residue once MetAP cleaves off the (previously deformylated) N-terminal Met (Fig. 1B). The N-terminal sequence fMet Thr (fMT) of a nascent D2^T2^ protein is a favorable sequence context for the PDF mediated deformylation of fMet, as had been shown, in particular, in a detailed study of substrate preferences of *E. coli *PDF for amino acid residues downstream from fMet 17.

When Giglione and colleagues 109,110 carried out pulse-chases to monitor the degradation of the wild-type D2^T2^ protein in chloroplasts, they found this protein to be relatively long-lived under normal conditions. However, D2^T2^ became short-lived in the presence of actinonin, which inhibited the PDF-mediated deformylation of nascent D2^T2^. To address the reason for this effect, Giglione *et al. *109,110 mutated Thr at position 2 of D2^T2^ to either Asp (D) or Glu (E). The resulting mutant proteins D2^T2D^ and D2^T2E^ were short-lived in chloroplasts even in the absence of actinonin, i.e., in the absence of PDF inhibition 109,110. The deformylated N terminal Met of wild-type D2^T2^ was expected to be cleaved off by MetAP, because Thr is smaller than Val (see Introduction). In contrast, N terminal Met was expected to be retained in the mutant D2^T2D^ and D2^T2E^ proteins, inasmuch as both Asp and Glu are larger than Val. Therefore Giglione and colleagues interpreted the accelerated degradation of D2^T2D^ and D2^T2E^ (compared to D2^T2^) in chloroplasts as resulting from the retention of their deformylated N terminal Met, i.e., as the consequence of the inability of MetAP to remove deformylated Met from the N-termini of D2^T2D^ and D2^T2E^, in contrast to wild-type D2^T2^ 110.

However, our results (Fig. 3) suggest a different, formyl-based interpretation of the above D2 findings 110. This alternative interpretation ascribes the destruction of the mutant D2^T2D^ and D2^T2E^ proteins to a *relatively slow* PDF-mediated deformylation of N-terminal fMet if it is followed by either Asp or Glu, in comparison to the at least 10-fold faster deformylation of fMet if it is followed, for example, by the Thr residue, which is present at position 2 of wild-type D2^T2^. Thus, we suggest that the correct interpretation of the earlier data about the protein D2^T2^ is the one in which D2^T2^ can be degraded through its fMet/N degron if deformylation of fMet in D2^T2^ is inhibited by actinonin. Further, the data described below (Fig. 3E, F) suggest that the previously observed rapid destruction of the mutant D2^T2D^ and D2^T2E^ proteins 109,110 is also mediated by their fMet/N degrons, because the N-terminal fMet-Asp and fMet Glu sequences of D2^T2D^ and D2^T2E^ are the least favorable sequence contexts for the PDF mediated deformylation of N-terminal fMet, as had been shown in a detailed study of the sequence preferences of *E. coli* PDF 17.

The P1^T2^ protein (D2^1-11^ e^K^ ha Ura3) and the otherwise identical P1^T2D^ protein, with Asp replacing Thr at position 2, were expressed from the arabinose-inducible P*_ara_* promoter (Fig. 3A, B and Table S2). These reporters were identical, in their 11 residue N-terminal segments, to the N terminal sequences of the wild type D2^T2^ and mutant D2^T2D^ proteins that had been studied in the cited chloroplast based experiments 109,110. Two other plasmids expressed the otherwise identical Ub-P1^T2^ and Ub-P1^T2D^, i.e., the Ub-fusion counterparts of P1^T2^ and P1^T2D^ (Fig. 3C and Table S2).

Ub is not recognized as a degron in wild-type *E. coli*. However, a Ub fusion can be cotranslationally cleaved in *E. coli* if they express a deubiquitylating (DUB) enzyme such as Ubp1 of *S. cerevisiae* 75,113,114. Placing the Ub moiety in front of two reporters and expressing the resulting Ub fusions in Ubp1-containing *E. coli* allowed the production of P1^T2^ and P1^T2D^ through the site-specific removal, by Ubp1, of the N terminal Ub moiety. (This version of the Ub fusion technique was developed in our studies of the *E. coli* N-end rule pathway 75,113,114.) The difference between two modes of reporter expression, the direct one and the Ub fusion mediated one, is the transient presence of the fMet residue at the N-termini of directly produced P1^T2^ and P1^T2D^ vs. the presence of *unformylated* N-terminal Met in the otherwise identical P1^T2^ and P1^T2D^ that had been generated from Ub-P1^T2^ and Ub P1^T2D^ through the removal of their Ub moiety (Fig. 3C). It should be noted that although N terminal fMet was present at the N-terminus of nascent Ub upon the expression of Ub-P1^T2^ and Ub-P1^T2D^ in *E. coli*, the rapid folding of the emerging Ub moiety would be expected to facilitate deformylation of its N-terminal fMet, thereby abrogating its fMet/N-degron (Fig. 1D).

Equal total protein loads were controlled as described above for P1^T2^ in wild-type and *fmt* cells (Fig. 3D). Extracts from wild-type *E. coli* containing the directly expressed P1^T2^ and P1^T2D^ reporters were fractionated by SDS PAGE, followed by immunoblotting with anti ha antibody (Fig. 3E, F). Whereas the band of P1^T2^ could be detected in cells growing in the presence of arabinose, the level of the otherwise identical P1^T2D^, containing Asp at position 2 (this is an unfavorable sequence context for fMet deformylation 17) was either too low for detection in one experiment (Fig. 3F, lanes 1-4) or was detectable but considerably lower than the level of P1^T2^ in another, independent experiment (Fig. 3E, lanes 1-4).

In contrast, when the same two reporters, P1^T2^ and P1^T2D^, were expressed as Ub fusions in *E. coli* that also expressed the yeast Ubp1 DUB enzyme, two changes were observed. First, the yields of both reporters were greatly increased. Second, their steady-state levels, instead of being strongly different upon reporters’ direct expression, became equal (Fig. 3C, E, F). Expression of the same Ub fusions in *E. coli* lacking the Ubp1 DUB yielded equal levels of the larger, unprocessed Ub-P1^T2^ and Ub-P1^T2D^ fusions (Fig. 3F, lanes 9-12).

These findings (Fig. 3B, C, E, F), together with the data about P1^T2^ in wild type vs. *fmt* *E.coli* (Figs. 3A, D), suggested that the N-terminal fMet residue of both nascent and just completed, newly formed proteins can participate in two alternative transitions: the PDF-mediated deformylation of N terminal fMet vs. its capture by the postulated fMet/N recognin/protease and the ensuing processive degradation of a targeted protein (Fig. 1C E and Discussion).

### Formylation-dependent selective destabilization of a reporter protein.

In these ^35^S pulse chase assays, our reporters were derivatives of a natural cytosolic *E. coli* protein, the 164-residue PpiB peptidyl-prolyl cis-trans isomerase 115. One feature of these assays (Fig. 4) was a “built-in” reference protein. fMVTF, the N terminal sequence of PpiB, is a motif favored by the *E. coli* PDF deformylase 17. This sequence is indicated by the superscript on the left side of the term ^MVTF^_wt_PpiB_f _, which denotes the C terminally flag-tagged wild-type PpiB, a reference protein. It was coexpressed with one of two otherwise identical reporters, termed, respectively, ^MYFY^PpiB_f_ Ub and ^MDDD^PpiB_f_-Ub (Fig. 4). The reference protein ^MVTF^_wt_PpiB_f _ and the reporter ^MYFY^PpiB_f_-Ub were coexpressed from two identical, arabinose-inducible, tandemly arranged P*_ara_* promoters (Fig. 4A, B). An otherwise identical plasmid coexpressed the reference _f_ and the reporter ^MDDD^PpiB_f_ Ub (Fig. 4C, D and Table S2).

The two reporters, ^MYFY^PpiB_f_ Ub and ^MDDD^PpiB_f_ Ub, differed from the reference ^MVTF^_wt_PpiB_f _ at two places: by the sequence of three residues following N terminal Met and by the presence of the ~8 kDa Ub moiety C terminally to the PpiB_f_ moiety (Fig. 4A, C). The Ub moiety was linked, C-terminally, to the PpiB moiety solely for making it easy to distinguish, by SDS PAGE, the resulting reporters ^MYFY^PpiB_f_ Ub and ^MDDD^PpiB_f_ Ub from the reference ^MVTF^_wt_PpiB_f _ (Fig. 4).

Three residues, Val Thr Phe, which follow N-terminal Met in wild-type PpiB, were replaced, in ^MYFY^PpiB_f_-Ub and in ^MDDD^PpiB_f_-Ub, by the sequences Tyr-Phe-Tyr (YFY) and Asp Asp-Asp (DDD), respectively (Fig. 4). ^MYFY^PpiB_f_ Ub was our “rapidly deformylatable” reporter (also called reporter-1), since N-terminal sequences of the kind exemplified by the sequence fMYFY are kinetically favorable contexts for the PDF mediated deformylation of N terminal fMet (Fig. 4A) 17. The N terminal sequence fMVTF, of the reference _f_, is also a favorable motif for the PDF mediated deformylation of N terminal fMet 17.

The encoded N-terminal sequence of ^MDDD^PpiB_f_-Ub (reporter-2) was Met Asp Asp-Asp (MDDD) (Fig. 4C). ^MDDD^PpiB_f_-Ub was our “slowly deformylatable” reporter, because the N terminal sequence fMDDD has been shown to be among the most unfavorable contexts for the PDB mediated deformylation of N terminal fMet 17. Deformylation, by purified *E. coli* PDF, of synthetic peptides bearing N terminal fMet was at least 10-fold faster for most favorable fMet sequence contexts, in comparison to least unfavorable ones 17. These sequence motifs were exemplified, in our reporters, by fMYFY and fMVTF (favorable contexts) vs. fMDDD (unfavorable context) (Fig. 4A, C).

If fMet/N-degrons exist (in other words, if the postulated fMet/N-recognin/protease exists), the relatively slowly deformylated reporter-2 and the relatively rapidly deformylated reporter-1 would be vulnerable both to the PDF-mediated deformylation of their N-terminal fMet (a step that abrogates fMet/N-degrons) and to the alternative, competing event of capture and processive degradation of a reporter protein through its fMet/N-degron. In the latter outcome, the postulated fMet/N recognin/protease succeeds in binding to N terminal fMet before its deformylation by PDF. Either one of these mutually exclusive steps would take place while the polypeptide chain of a nascent reporter continues to emerge from the ribosomal tunnel at the rate of 10 20 residues/sec.

Given this disposition, one key prediction of the fMet/N-degron hypothesis is as follows: if the N-terminal fMet residue of one nascent protein (reporter-2) is deformylated significantly slower than fMet of another (nearly identical) nascent protein (reporter-1) (Fig. 4A, C), the molecules of reporter-2 would be targeted for destruction more often through its (more frequently retained) fMet/N-degron, resulting in a higher rate of degradation of reporter-2.

Because these events are expected to involve largely nascent, still growing polypeptide chains, the second prediction is that a difference in degradation rates between reporter-1 and reporter-2 in wild-type *E. coli* may be largely confined to previously glimpsed proteolytic processes referred to as the “time-zero”, “before-chase” proteolysis 67,68,69,71,97,116,117,118. These effects result from the processive *cotranslational* degradation, in contrast to *posttranslational* degradation. While the posttranslational degradation of a protein is measured during a chase, the extent of cotranslational degradation of the same protein is determined by comparing time-zero (before chase) levels of this protein and an otherwise identical protein that lacks (or nearly lacks) the relevant degron 67,68,69,71,97,116,117,118. Given the second prediction above, the presence, in our assays, of the “built-in” reference protein ^MVTF^_wt_PpiB_f _ (Fig. 4) was particularly important, because a reference greatly increases the accuracy of quantifying both cotranslational and posttranslational degradation, with the cotranslational mode revealing itself through time-zero, before-chase effects.

The third and equally critical prediction of the fMet/N-degron hypothesis: if a faster degradation of the more slowly deformylated reporter 2 is actually observed in wild type cells (possibly as a time-zero, before-chase effect), this effect should vanish if ^35^S-pulse-chases are performed in *fmt* cells, which lack formyltransferase and therefore lack fMet/N-degrons.

Experiments designed as described above were carried out. The results were in agreement with all three predictions of the fMet/N-degron hypothesis (Fig. 4).

In the first set of ^35^S-pulse-chases, wild-type *E. coli* and its *fmt* mutant (lacking Met formylation) were transformed with pKP458, which expressed, from two identical P*_ara_* promoters, the rapidly deformylatable _f_ reference and the also rapidly deformylatable ^MYFY^PpiB_f_ Ub reporter-1 (Fig. 4A, B and Table S2). Arabinose was added to induce expression of the two proteins, followed by 1-min pulse with ^35^S methionine/cysteine at 37°C, chases for 1, 3, 7, 15 and 30 min, preparation of cell extracts, immunoprecipitation of ^MVTF^_wt_PpiB_f_ and ^MYFY^PpiB_f_ Ub with anti-flag antibody, fractionation of precipitated proteins by SDS PAGE, and autoradiography. The data were quantified by plotting, on a semi logarithmic scale, the ratios of ^35^S in the band of the ^MYFY^PpiB_f_ Ub reporter-1 to ^35^S in the band of the reference ^MVTF^_wt_PpiB_f_ (Fig. 4A, B, E).

^MVTF^_wt_PpiB_f _ and ^MYFY^PpiB_f_ Ub were relatively stable over the 30-min chase in wild-type and *fmt* *E. coli*. In addition, no significant differences in ^35^S ratios of ^MYFY^PpiB_f_ Ub to ^MVTF^_wt_PpiB_f _ at the time-zero (before chase) point were observed between wild-type and *fmt* cells (Fig. 4E; curves 1 and 2). That was expected, given the approximately equal rates of the PDF-mediated deformylation of the reference and reporter-1, as described above.

In the second set of ^35^S-pulse-chases, wild-type and* fmt* *E. coli* were transformed with pKP459, which expressed, from the two P*_ara_* promoters, the rapidly deformylatable ^MVTF^_wt_PpiB_f _ and the relatively slowly deformylatable^ MDDD^PpiB_f_-Ub reporter-2 (Fig. 4C, D and Table S2). This comparison revealed a strikingly high ~8-fold difference between the rates of time-zero, before chase degradation of the rapidly deformylatable ^MYFY^PpiB_f_-Ub reporter-1 and the relatively slowly deformylatable ^MDDD^PpiB_f_ Ub reporter-2, indicating a much higher rate of the early, presumably cotranslational degradation of the (relatively) slowly deformylated ^MDDD^PpiB_f_-Ub in wild-type cells (Fig. 4E; curves 2 and 4). Crucially, the bulk of this effect was abrogated when the otherwise identical ^35^S-pulse-chases were performed in *fmt* cells, which did not formylate N terminal Met and therefore could not create fMet/N-degrons (Fig. 4E; curves 1 and3).

While the degradation of the ^MDDD^PpiB_f_-Ub reporter-2 in wild-type cells was largely of the time-zero, before-chase kind, this reporter was also destroyed, relatively slowly, during chases in both wild-type and *fmt* cells (Fig. 4C-E; curves 3 and 4). In contrast, little posttranslational degradation was observed with the ^MYFY^PpiB_f_-Ub reporter (differing from ^MDDD^PpiB_f_-Ub by three residues after N terminal Met) in either wild-type or *fmt* cells (Fig.4A,B, E). A parsimonious interpretation of the slow posttranslational degradation of ^MDDD^PpiB_f_-Ub is that the sequence of three Asp residues after N terminal Met may have created a weak, largely posttranslational and formylation-unrelated degron.

The design of these assays, i.e., their built-in reference protein as well as two identical transcriptional promoters expressing a reference and a reporter in wild-type vs. *fmt* cells, controlled for variables other than protein degradation (Fig. 4A, C). The ~8-fold difference in the time-zero, before-chase levels of the rapidly deformylated reporter-1 and the relatively slowly deformylated reporter 2 in wild-type cells, and the dependence of this effect on the presence of formyltransferase (Fig. 4E) seem to allow only one plausible and parsimonious interpretation. Specifically, we posit that this difference and its dependence on Nt-formylation indicated the presence, in our reporters, of PDF-vulnerable fMet/N-degrons (Figs. 1C-E and 4).

## DISCUSSION

Key results of the present study are the evidence that rapidly and slowly deformylated protein reporters are destroyed at different rates in wild-type *E. coli*, and that this effect is abrogated in formylation-lacking *fmt* mutants. These and related findings strongly suggest that the formylated N terminal fMet residue can act as an N-degron, termed fMet/N-degron, of a novel bacterial N-end rule pathway, termed the fMet/N-end rule pathway (Figs. 1-4).

### Incomplete deformylation of nascent bacterial proteins *in vivo*

N-terminal fMet of nascent polypeptides can be incompletely deformylated by PDF *in vivo* 60,119. The incomplete deformylation is particularly pronounced with DNA-encoded, ribosome-generated small natural peptides 120. For example, the bulk of secreted 7 residue microcin-C peptide is not deformylated *in vivo* 120, although this peptide’s second residue, in the N-terminal sequence fMet-Arg, is one of position-2 residues that are optimal for deformylation of fMet by PDF 17.

The molar concentration of PDF in *E. coli* is 2-3 μM, an order of magnitude below that of the ribosomes, ~30 μM 7,23,37. Consequently, an efficacious deformylation of nascent proteins requires that molecules of PDF “hop” among the PDF-binding sites of different ribosomes. Given the resulting stochasticity of deformylation, given low steady-state levels of PDF in the bulk solvent (since most PDF molecules are ribosome-bound), and given a significant dependence of the rate of deformylation by PDF on fMet-proximal sequence contexts 17,18, one should expect an incomplete deformylation of bacterial proteins to be a frequent occurrence 60,119. For example, a 2 D electrophoretic study of abundant *Bacillus subtilis* proteins indicated that some of them retained, at steady-state, a small but significant fraction of their initial (formylated) N terminal fMet 121.

Analyses, using 2-D electrophoresis, of the *in vivo* inhibition of fMet deformylation by the PDF-specific inhibitor LBM-415 in *Staphylococcus aureus* and *Streptococcus pneumoniae* demonstrated the accumulation of formylated (non-deformylated) counterparts of normally deformylated proteins 122. Interestingly, while a subset of proteins in LBM-415-treated bacterial cells exhibited a telltale double-spot appearance on 2-D gels (a formylated plus deformylated species), many other proteins remained as single spots, without formylated counterparts 122. This finding, remarked upon but not explained by the authors 122, might signify the selective degradation of some formylated proteins through fMet/N-degrons suggested by the results of our study (Figs. 1-4). In this interpretation of the data in ref. 122, those proteins that accumulate, in the presence of PDF inhibitor, as formylated (non deformylated) species, might be partially protected from degradation owing to a steric shielding (sequestration) of their fMet/N degrons, either through intramolecular protein folding or through the formation of “protective” oligomeric complexes with cognate protein ligands. The latter mechanism would be analogous to the previously discovered shielding mediated conditionality of natural eukaryotic Ac/N degrons 68.

### Working model of fMet/N-degrons

The idea of fMet/N-degrons was sketched in ref. 67. It is now described in detail (Fig. 1C-E) vis-á-vis the data (Figs. 2-4).

First, we presumed that a distinct fMet/N-recognin/protease (envisioned as a transient complex of both) can recognize, in a competition with PDF, the N-terminal fMet moiety of a nascent protein and thereby initiate a processive destruction of this protein either cotranslationally or posttranslationally. The latter distinction is based on whether the protein’s N terminal fMet is captured by the fMet/N-recognin/protease before or after the protein’s nascent polypeptide chain is released from the last tRNA molecule at the ribosome’s peptidyltransferase site. Cotranslational protein degradation is defined as the processive degradation of a nascent, growing polypeptide that exists, at the time of proteolytic attack, as a ribosome-associated peptidyl-tRNA.

A proteolytic pathway that targets a specific degron in a protein and converts the bulk of it to short peptides must be highly processive. A nonprocessive protease would tend to release an initially captured protein and thereby would lose it for good if a protein’s segment containing the degron had already been destroyed. The postulated bacterial fMet/N-recognin/protease is envisioned to be a processive proteasome-like protease, possibly one of the known ones, such as, for example, FtsH, Lon, or a ClpP-containing protease 123 (see also Concluding Remarks). One protease of the latter class, ClpXP, is unlikely to be involved, because its *in vitro* activity toward a test protein was shown to be independent of the presence or absence of Nt formylation 22.

The degradation of an fMet-bearing nascent protein, i.e., of a ribosome-bound peptidyl tRNA, would proceed to completion once it begins, after the recognition of protein’s N terminal fMet. During this (postulated) degradation, the fMet/N-recognin/protease, having captured the protein’s N terminal fMet, would remain associated with the translating ribosome. The emerging chain of a nascent protein would continue to be delivered into the protease’s chamber and destroyed to short peptides until the natural (i.e., not premature) chain termination event at the ribosome’s peptidyl transferase site. An alternative possibility is that the initiation of cotranslational degradation of a ribosome-associated peptidyl-tRNA would lead, through allosteric effects, to a premature termination of translation.

Second, the fMet/N recognin/protease or at least its fMet/N-recognin part was presumed to have a non zero affinity for the ribosomes, forming a “cloud” of fMet/N-recognin/protease molecules (or fMet/N-recognin alone) “hugging” the ribosomes. The analogous cloud of ribosome-hopping PDF molecules 23 would partially overlap with the (presumed) cloud of fMet/N recognin/protease molecules. One version of this model envisions a “tighter cloud” of ribosome-hopping PDF molecules, i.e., a smaller time-averaged distance between them and the ribosomes, in comparison to a “looser cloud” of fMet/N-recognin/protease molecules, reflecting their (presumed) lower affinity for the ribosomes. In such a setting, which was partly characterized for PDF 23 and is postulated here for the fMet/N recognin/protease (or its fMet/N-recognin part), a molecule of ribosome-bound PDF would have a stochastically better “shot” at binding to and deformylating N-terminal fMet of an emerging nascent protein. The term “looser cloud” implies a larger time-averaged distance of the fMet/N recognin/protease from the tunnel’s exit, in comparison to PDF. Whether the “cloud” model is relevant to a postulated fMet/N-recognin rather than to a “downstream” protease remains to be seen, particularly if the protease in question is the inner membrane-embedded FtsH protease (see Concluding Remarks).

In this working model of fMet/N-degrons, some non-wild-type N terminal sequences, once they emerge from the ribosomal tunnel, would either not collapse rapidly enough, or would collapse into globules that impede deformylation of N-terminal fMet by PDF. In the latter case, a collapse may prevent, at least in part, an exposure of the roughly 10-residue N terminal region (including its fMet) on the globule’s surface. As a result, the ribosome-bound PDF would often fail to deformylate fMet, given the narrow kinetic/stochastic window of opportunity that PDF is allowed to have (Fig. 1C-E). As to the former case, the radii of gyration of folded polypeptides with lengths of up to 100 residues are 1.0-1.2 nm, whereas the radii of gyration of unfolded polypeptides increase from ~1.0 to ~3.0 nm as their length increases from 8 to 100 residues 124. In such a setting, the N-terminal fMet residue of a (largely) unfolded polypeptide may be stochastically and partially buried in a fluctuating, partially folded conformation, thereby impeding the capture and deformylation of fMet by the ribosome-bound PDF (Fig. 1C-E).

Results of a study based on the ribosome profiling technique suggested that the PDF mediated deformylation of nascent bacterial proteins takes place, in the main, before they become significantly larger than ~100 residues 28. Thus, the postulated targeting of a nascent Nt formylated protein for processive degradation through its fMet/N-degron (Fig. 1C-E) may be “decided upon” largely on the basis of protein’s first 100 or so residues. In other words, significantly more distal regions of a protein may often not be involved.

Given this disposition, we suggest that ~100-residue N-terminal regions of bacterial proteins evolve under a selection pressure that tends to maximize their ability to collapse into a “molten globule” 125 in which roughly 10 N-terminal residues, including N terminal fMet, tend to be extruded from the globule and exposed to solvent. Consequently, PDF would be able to deformylate a nascent protein before the N-terminal fMet residue would move too far away from the ribosome-bound PDF, owing to the ongoing elongation of the protein’s polypeptide chain (Fig. 1C, D). Entries in Protein Data Bank (http://www.rcsb.org/pdb/home/home.do) exhibit a tendency for N-terminal regions to be weakly ordered in crystal structures. Moreover, such regions are often absent altogether in published structures, having been removed from the proteins’ natural N-termini to allow crystallization. For example, the first high-resolution structures of “soluble” eukaryotic proteins with intact natural N terminal regions (usually in complexes with their cognate protein ligands) have been determined only recently 126,127,128.

Third, the kinetic advantage of PDF in targeting the N-terminal fMet residue would be transient, because a stochastic failure of PDF to capture and deformylate fMet of a nascent protein would soon (within seconds) position that fMet outside the physical reach of a ribosome bound PDF molecule, owing to the ongoing elongation of the protein’s polypeptide chain. It would be, then, the stochastic and also transient turn of the less tightly ribosome-bound fMet/N recognin/protease or its fMet/N-recognin part (resulting in its larger time-averaged distance from the ribosomes) to capture the N-terminal fMet residue, whose distance from the tunnel’s exit may continue to increase as the nascent polypeptide keeps emerging from the ribosome.

Fourth, the main (but not necessarily the sole) function of postulated fMet/N-degrons (Fig.1E) is envisioned to be the quality control of nascent bacterial proteins and just released, newly formed proteins. This role of bacterial fMet/N degrons would be similar to the previously identified quality-control function of eukaryotic Ac/N-degrons 67,68,69,70. The naturally high (~10^ 3^ per residue) frequency of amino acid misincorporation during protein synthesis can be further increased by antibiotics that elevate the ribosome’s error rate. Such antibiotics are endemic in natural bacterial habitats 7,51. On the assumption that the error rate is approximately uniform along the sequence of a translated polypeptide, a significant fraction of N-terminal regions of nascent proteins would be mutant vis á-vis their wild-type DNA-encoded sequences even in the absence of stress. The frequency of abnormal (mistranslated) sequences would be further elevated in the presence of fidelity decreasing antibiotics or other stresses.

Fifth, some molecules of just completed, newly formed proteins would stochastically and at least transiently bypass the targeting by both PDF and the postulated fMet/N-recognin/protease. The non-ablated fMet/N-degrons of such proteins are envisioned to be often rapidly repressed through their steric shielding (sequestration), owing to interactions of newly formed proteins with their cognate ligands, which can be other proteins, RNA or DNA. In contrast, a nascent or a newly formed Nt formylated protein that cannot form such “protective” complexes efficaciously enough or cannot form them at all (owing, for example, to its misfolding) would remain vulnerable to either the destruction by the postulated fMet/N recognin/protease or to the PDF-mediated deformylation of N-terminal fMet, a step that abrogates the protein’s fMet/N-degron. This model presumes a low level of PDF away from the ribosomes, in agreement with experimental evidence 23. In contrast, the postulated fMet/N recognin/protease (or its fMet/N-recognin part), while also ribosome-associated, is presumed to be present at significant levels in the bulk solvent as well.

Studies by Green and colleagues employed defined *in vitro* translation systems and showed that a bacterial (but apparently not eukaryotic) ribosome can sense a misincorporation of a non cognate residue during protein synthesis and react through a further decreased fidelity of translation downstream from the incorrect amino acid residue 129,130. This error-induction response increases the probability of premature translation termination and the release of a mistranslated nascent polypeptide 129. The extent of Green’s effect remains to be determined *in vivo*. If the frequency of nascent polypeptides that are prematurely terminated in living bacteria owing to this effect is as high as it was observed to be *in vitro* 129, one would expect a significant frequency of Nt-formylated, mistranslated and prematurely terminated proteins that emerge from the ribosomal tunnel while bearing fMet/N-degrons. Owing to misincorporation events that led to their premature release, such (incomplete) proteins would often fold either abnormally or not at all. These properties may render them less susceptible to deformylation.

The temporal and geometric aspects of N-terminal fMet vis-á-vis other participants in this kinetic drama would vary from one nascent protein to another, at least in part because the rate of PDF-mediated deformylation of fMet in a nascent protein depends on the identities of residues at position 2 and beyond 17. In sum, the folding (or misfolding) of a growing nascent protein, and the propensity (or its absence) of N terminal fMet to remain sterically accessible to PDF on the surface of a nascent protein globule would affect the outcomes of competition between the ribosome-associated PDF and the postulated fMet/N recognin/protease. This glimpse of possible mechanics is an illustration of complexities that remain to be understood vis-á-vis the concept of fMet/N-degrons (Fig. 1C-E).

## CONCLUDING REMARKS

The fMet/N-degron hypothesis was proposed in 2010 67. The difficulty in viewing (let alone proving) the N-terminal fMet residue as a degradation signal stems from the transiency of the formyl group of N terminal fMet in a majority of nascent bacterial proteins.

The rate of chain elongation by bacterial ribosomes *in vivo* at 37°C is 10-20 residues/sec, i.e., it is up to an order of magnitude higher than estimated rates of chain elongation by the cytosolic ribosomes in eukaryotes 49,50,51,52. Faster emergence of nascent proteins from bacterial ribosomes may have precluded the adoption, during bacterial evolution, of cotranslationally (as distinguished from pretranslationally) created N degrons, such as, for example, Ac/N-degrons in eukaryotes (Fig. S1B). Notably, the Nt acetylation of many eukaryotic proteins is known to be incomplete 103, i.e., the cotranslational generation of Ac/N degrons is often not efficacious enough even at relatively low rates of chain elongation in eukaryotes. This fact is consistent with the view that the observed pervasiveness of the pretranslational formylation of N terminal Met (all examined wild-type bacteria contain fMet) resulted from selection pressures to maximize the extent of Met formylation vis-á-vis high rates of polypeptide chain elongation.

Competition among bacteria and other microorganisms often involves antibiotics that increase the frequency of translational errors in susceptible strains. One function of fMet/N degrons is envisioned to be the preferential degradation of misfolded nascent proteins. Thus, stresses caused by perturbed translation, including antibiotics-mediated conflicts in the microbial world, may be a source of selection pressures that retained the apparatus of bacterial fMet/N-degrons.

Now that the first evidence for fMet/N-degrons has been produced (Figs. 1-4), the next essential step is to identify the postulated, possibly two-component fMet/N recognin/protease. A recent study by Bittner *et al. *131 described the N-terminal degradation signal of YfgM, an inner membrane-embedded *E. coli* protein. Analyses by Bittner *et al.* 131 did not invoke either an fMet/N-degron or the formylation of N-terminal Met. However, specific properties of the cytosol facing N-terminal degron of YfgM 131 suggested, to us, that this degradation signal may be an fMet/N-degron. If so, the inner membrane-embedded, ATP dependent FtsH protease would be the one that targets the N-terminal fMet residue (either directly or through an unknown fMet/N recognin), because Bittner *et al.* 131 identified FtsH as the protease that destroys YfgM. Remarkably, our recent studies showed that the degron of YfgM is, in fact, an fMet/N-degron, thereby identifying FtsH as the relevant protease (T.T.M.V., K.P. and A.V., unpublished data).

## MATERIALS AND METHODS

### Miscellaneous reagents

Anti-flag M2 Magnetic Beads (M8823), anti-flag M2 antibody, and anti-ha antibody were from Sigma-Aldrich. Complete EDTA-free Protease Inhibitor Cocktail Tablets were from Roche. Express [^35^S]Protein Labeling Mix (1.175 Ci/mmol) was from Perkin Elmer. Methionine/cysteine-free synthetic complete ("Hopkins") supplement mixture (SC) was from Sunrise Science Products. Actinonin was from Enzo Life Sciences.

### Bacterial strains and mutagenesis

*E.* *coli* strains (Table S1) were grown at 37°C on Luria-Bertani (LB) medium. When used for selection, antibiotics were added to the following final concentrations: kanamycin (Km): 50 μg/ml; ampicillin (Amp): 100 μg/ml. Null *fmt* and *def-fmt* *E.* *coli* mutants (strains KPS73-KPS76) (Table S1) were constructed using a gene disruption strategy 132. The resulting *E. coli* mutants were grown in LB under selective conditions. The desired deletions were verified by polymerase chain reaction (PCR), followed by sequencing of PCR-amplified, purified DNA fragments.

### Construction of plasmids

*E.* *coli* DH5α (Invitrogen) was used for cloning and maintaining plasmids. Phusion High Fidelity DNA polymerase (New England Biolabs) was used for PCR. Specific DNA constructs were generated using standard techniques 112 and verified by DNA sequencing.

The plasmids containing one or two P*_ara_* promoters were derived from the pBADET vector, a gift from Dr. V. Ksenzenko (Institute of Protein Research, Pushchino, Russia). The plasmids pKP249 and pKP250, which expressed P1^T2X^ (P1^T2X^-e^K^-ha-Ura3) fusion proteins (X=Thr or Asp) from the P*_ara_* promoter, were constructed by subcloning a *Nde*I/*Hind*III-digested DNA fragment (produced by PCR from pCH178; Table S2) into the *Nde*I/*Hind*III-cut plasmid pBADET (Table S2). (The DNA fragment from pCH178 that encoded e^K^-ha-Ura3 was extended, by PCR, to yield fragments encoding either P1^T2^ e^K^ ha-Ura3 or P1^T2D^-e^K^ ha-Ura3.) To construct pKP251 and pKP252, which expressed Ub-P1^T2X^ fusion proteins from the P*_ara_* promoter, a DNA fragment containing the ORF of Ub was PCR-amplified (using the pCH178 plasmid as a template), digested with *NdeI*/*Bsp*EI and subcloned into* NdeI*/*Bsp*EI-cut pKP249 and pKP250 (Table S2). To construct pKP257 and pKP258, which expressed P1^T2X^ proteins (P1^T2X^-e^K^-ha-Ura3) (X=Thr or Asp) from the P*_KmR_* promoter, *Nde*I/*Hind*III-digested fragments, produced by PCR from pKP249 and pKP250, were subcloned into *Nde*I/*Hind*III-cut pACYC177 (Table S2). The plasmids pKP286 and pKP287, which expressed^ MXXX^PpiB His_8_-flag (^MXXX^PpiB_f_) proteins (XXX=Val Thr-Phe or Asp-Thr-Phe), were constructed by subcloning a *Nde*I/*Xba*I-digested DNA fragment (encoding PpiB and produced by PCR from MG1655 *E. coli *genomic DNA) into *Nde*I/*Xba*I-cut pBADET (Table S2). The plasmids pKP458 and pKP459, each of which expressed two PpiB-derived proteins from two identical P*_ara_* promoters (Fig. 4) were constructed as follows. DNA fragment containing the P*_ara_* promoter was PCR-amplified from pBADET (Table S2). DNA fragment encoding ^MXXX^ppiB-His_8_-flag-Ub (^MXXX^PpiB_f_-Ub) (XXX=Tyr Phe Tyr or Asp-Asp-Asp) was PCR-amplified from pKP335 (Table S2). The resulting DNA fragments were linked together using PCR, digested with *Afe*I/NsiI and thereafter subcloned into *Afe*I/NsiI-cut pKP286 (Table S2). Additional cloning details are available on request.

### Immunoblotting assays

Methods for data in Fig. 3D

Wild-type and mutant* E.* *coli* (CAG12184, KPS73 and KPS74; Table S1) carrying pKP257 (Table S2) were grown at 37°C overnight in Growth Medium (GM) (M9 medium (33.9 mg/ml Na_2_HPO_4_, 15 mg/ml KH_2_PO_4_, 5 mg/ml NH_4_Cl, 2.5 mg/ml NaCl, pH 7.0), 0.5% glycerol, 0.2% glucose, 40 μg/ml Met, 40 μg/ml Cys, methionine/cysteine-free synthetic complete (SC) mixture (Sunrise Science Products) supplemented with ampicillin (Amp; 50 µg/ml)). Cultures were diluted 1:100 in 30 ml of GM medium and incubated on a shaker at 37°C until A_600_ of ~0.5. The resulting cultures (10 ml) were centrifuged at 5,000 *g* for 5 min at 4°C, washed three times with 1-ml samples of ice-cold phosphate-buffered saline (PBS) and thereafter lysed in 0.1 ml volumes of 1% SDS. The resulting extracts were clarified by centrifugation at 14,000 *g* for 5 min at 4°C, and protein concentration in the supernatants were determined using Pierce BCA Protein Assay (Fisher Scientific). Samples were mixed with equal volume of 2 x SDS-sample buffer and heated at 95°C for 10 min. 25 μg of total protein in the resulting samples were subjected to SDS 4 12% NuPAGE (Invitrogen), followed by immunoblotting, using standard procedures 67,68 with a monoclonal anti ha antibody (1:2,000) (Sigma-Aldrich), with detection using ECL Plus (GE Healthcare).

Methods for data in Fig. 3E, F

Wild-type *E.* *coli* CAG12184 (Table S1) carrying pKP249 252, and pJT184 (Table S2) were grown at 37°C overnight in GM medium as described above. Cultures were diluted 1:100 in 30 ml of GM medium and incubated at 37°C until A_600_ of ~0.35. Cells were pelleted by centrifugation 5,000 *g* for 5 min at room temperature (RT), washed with 1 ml of Induction Medium-Ara-0.25 (IM-Ara-0.25) (M9 medium (pH 7.0), 0.5% glycerol, 0.25% arabinose, 40 μg/ml Met, 40 μg/ml Cys, methionine/cysteine-free synthetic complete (SC) mixture) and resuspended in 30 ml of IM-Ara-0.25 medium. After 90 min of incubation at 37°C, the resulting cultures (10 ml) were centrifuged at 5,000 *g* for 5 min at 4°C, washed three times with 1-ml samples of ice-cold PBS and thereafter processed for lysis, SDS-PAGE, and immunoblotting with anti-ha antibody as described above.

### Pulse-chase assays without immunoprecipitation

Methods for data in Fig. 2A 

Wild-type *E.* *coli* CAG12184 cells (Table S1) were grown in LB medium at 37°C overnight. 0.75 ml of overnight culture in LB was washed with 1 ml of GM medium, resuspended in 30 ml of GM, and was grown until A_600_ of ~0.35. Cells were pelleted by centrifugation at 5,000 *g* for 5 min at RT, washed with 1 ml of pre-warmed IM-Ara-0.25 medium and resuspended in 30 ml of pre-warmed IM-Ara-0.25. After 90 min of incubation on a shaker at 37°C in IM-Ara-0.25, 15 ml of the culture were centrifuged at 5,000 *g* for 5 min at RT, and washed 2 times with 1-ml samples of pre-warmed Pulse Medium-025 (PM Ara-0.25), which differed from the IM Ara 0.25 medium by lacking Met and Cys. Cell pellet was resuspended in 1 ml of PM Ara-0.25 and divided into 4 equal samples, which were incubated for 10 min at 37°C with agitation. Actinonin was added (to the final concentrations indicated in panels of Fig. 2A, B) at the beginning of 10 min incubations and was kept at the same concentrations throughout pulse-chases. Thereafter the cultures were labeled with 15 µl of Express [^35^S] Protein Labeling Mix (1.175 Ci/mmol, Perkin Elmer) for 1 min at 37°C, followed by centrifugation at 14,000 *g* for 30 sec at RT. Each supernatant was added to 0.3 ml of Chase Medium (CM) (M9 medium (pH 7.0), 0.5% glycerol, 0.5% glucose, 0.5 mg/ml Met, 0.5 mg/ml Cys_,_ 0.2 mg/ml chloramphenicol, methionine/cysteine-free synthetic complete (SC) mixture), followed by a chase, also at 37°C. Samples (0.1 ml) were withdrawn at indicated times during chase, followed by immediate freezing in liquid nitrogen. For further analyses, one volume of 2x SDS-PAGE sample buffer was added to a frozen sample, followed by heating at 95°C for 10 min, brief vortexing and centrifugation at 12,000 g for 5 min. 5 µl of each “time-zero” sample were spotted on Whatman 3MM filters, immersed in ice-cold 10% CCl_3_COOH for 5 min, boiled in 10 % CCl_3_COOH for 10 min, rinsed (for 15 sec) 3 times in 5% CCl_3_COOH, washed (for 5 min) 2 times with 5% CCl_3_COOH, rinsed 3 times with 95% ethanol, and air-dried, followed by measurements of ^35^S using Safety-Solve scintillation cocktail and scintillation spectrometer. 20,000 ^35^S cpm of each time-zero sample (Fig. 2B), and equal volumes of samples at later time points were subjected to SDS 4-12% PAGE, followed by autoradiography.

Methods for data in Fig. 2B

Wild-type (CAG12184) and *fmt* (KPS73) *E.* *coli* (Table S1) were grown in LB medium at 37°C overnight. Cultures were diluted 1:200 in the GM medium and incubated at 37°C until A_600_ of ~0.5. The resulting culture (7 ml) was centrifuged at 5000 *g* for 5 min at room temperature, washed three times with 1-ml samples of pre warmed PM-Ara-0.25 medium, and resuspended in 70 µl of PM-Ara-0.25, followed by incubation at 37°C for 10 min. A culture was labeled with 7 µl of Express [^35^S] Protein Labeling Mix (1.175 Ci/mmol, Perkin Elmer) for 1 min at 37°C. The labeling was quenched by the addition of 0.5 ml of CM (lacking chloramphenicol) and a chase, also at 37°C. Samples (0.1 ml) were withdrawn at indicated times during chase and mixed with 80 µl of TDS buffer (1% SDS, 5 mM dithiothreitol (DTT), 50 mM Tris-HCl, pH 7.4) containing “complete protease-inhibitor mixture” (Roche), followed by immediate freezing of samples in liquid nitrogen. Frozen samples were directly heated at 95°C for 10 min, and thereafter processed and analyzed identically to pulse-chase samples described above.

### Pulse-chase assays with immunoprecipitation

*E.* *coli* CAG12184, and pKP73 (Table S1) carrying pKP458 or pKP459 (Table S2) were grown at 37°C overnight in LB supplemented with Amp (50 µg/ml). Cultures were diluted 1:200 in fresh LB and grown until A_600_ of ~0.5. A resulting culture (7 ml) was centrifuged at 5000 *g* for 5 min at room temperature, washed three times with 1 ml samples of pre warmed PM-Ara-0.1 medium (containing 0.1% arabinose), and resuspended in 70 µl of PM-Ara-0.1, followed by incubation at 37°C for 10 min. Cultures were then pulse-labeled with 7 µl of Express [^35^S] Protein Labeling Mix (1.175 Ci/mmol, Perkin Elmer) for 60 sec at 37°C. The labeling was quenched by the addition of 0.5 ml of Chase-Medium (CM: M9 medium, pH 7.0, 0.5% glycerol, 0.25% glucose, 0.1 mM CaCl_2_, 2 mM MgSO_4,_ Methionine/Cysteine-free Synthetic Complete (SC) Mixture (Sunrise Science Products), 0.5 mg/ml unlabeled Met, 0.5 mg/ml unlabeled Cys). Chases were carried out also at 37°C. Samples (0.1 ml) were withdrawn at indicated times during chase and mixed with 80 µl of TDS buffer (1% SDS, 5 mM dithiothreitol (DTT), 50 mM Tris-HCl, pH 7.4) containing “complete protease-inhibitor mixture” (Roche), followed by immediate freezing of samples in liquid nitrogen. For further analyses, one volume of 2x SDS-PAGE sample buffer was added to a frozen sample, followed by heating at 95°C for 10 min. They were, thereafter, briefly vortexed and centrifuged at 12,000 g for 5 min. Supernatants were diluted with 10 volumes of TNN buffer (0.5 % NP40, 0.25 M NaCl, 5 mM Na-EDTA, 50 mM Tris-HCl (pH 7.4), containing “complete protease-inhibitor mixture” (Roche)), and processed for immunoprecipitation. 5 µl of each “time-zero” sample were spotted on Whatman 3MM filters and processed for measurements of CCl_3_COOH-insoluble ^35^S as described above. 5.5x10^6^ ^35^S cpm of each of the time-zero samples, and equal volumes of the following time points for each pulse-series were processed for immunoprecipitation, using magnetic beads with immobilized anti flag antibody M2 (Sigma; 7 µl of settled beads for each sample). The samples were incubated with rocking at 4°C for 3 h, followed by four washes of the beads in TNN buffer, resuspension of pellets in 20 μl of SDS-sample buffer, incubation at 95°C for 5 min, and the removal of beads. The resulting samples were fractionated by SDS-PAGE using NuPAGE 4 12% Bis-Tris gradient gels, followed by autoradiography and quantification using PhosphorImager (Molecular Dynamics, Sunnyvale, CA).

## SUPPLEMENTAL MATERIAL

Click here for supplemental data file.

All supplemental data for this article are also available online at http://microbialcell.com/researcharticles/formyl-methionine-as-a-degradation-signal-at-the-n-termini-of-bacterial-proteins/.
