# Supplementary material for: Formyl-methionine as a degradation signal at the N-termini of bacterial proteins
Source: Microb Cell. 2015 Sep 6;2(10):376–93. doi: 10.15698/mic2015.10.231 (PMC4745127; doi:10.15698/mic2015.10.231)
Supplement: Supplementary file 1 [file mic-02-376-s01.pdf]

Microbial Cell

Revised, August 13, 2015

Supplemental Materials for Research Article in *Microbial Cell*:

Formyl-methionine as a degradation signal at the N-termini of bacterial proteins

Konstantin I. Piatkov[1,3#], Tri T. M. Vu[1,#], Cheol-Sang Hwang[2], and Alexander Varshavsky[1,*]

[#]These authors contributed equally to this study.

[1]Division of Biology, California Institute of Technology, Pasadena, CA 91125, USA

[2]Department of Life Sciences, Pohang University of Science and Technology, Pohang, Gyeongbuk, 790-784, South Korea

[3]Center for Biotechnology and Biomedicine, Skolkovo Institute of Science and Technology, Moscow, 143026, Russia

**Corresponding author*: Alexander Varshavsky, Division of Biology and Biological Engineering, California Institute of Technology, Pasadena, CA 91125, USA. Telephone: 626-395-3785. Email: avarsh@caltech.edu

These Supplemental Materials contain:

Figure S1 and its legend.

Tables S1 and S2.

Supplemental References.

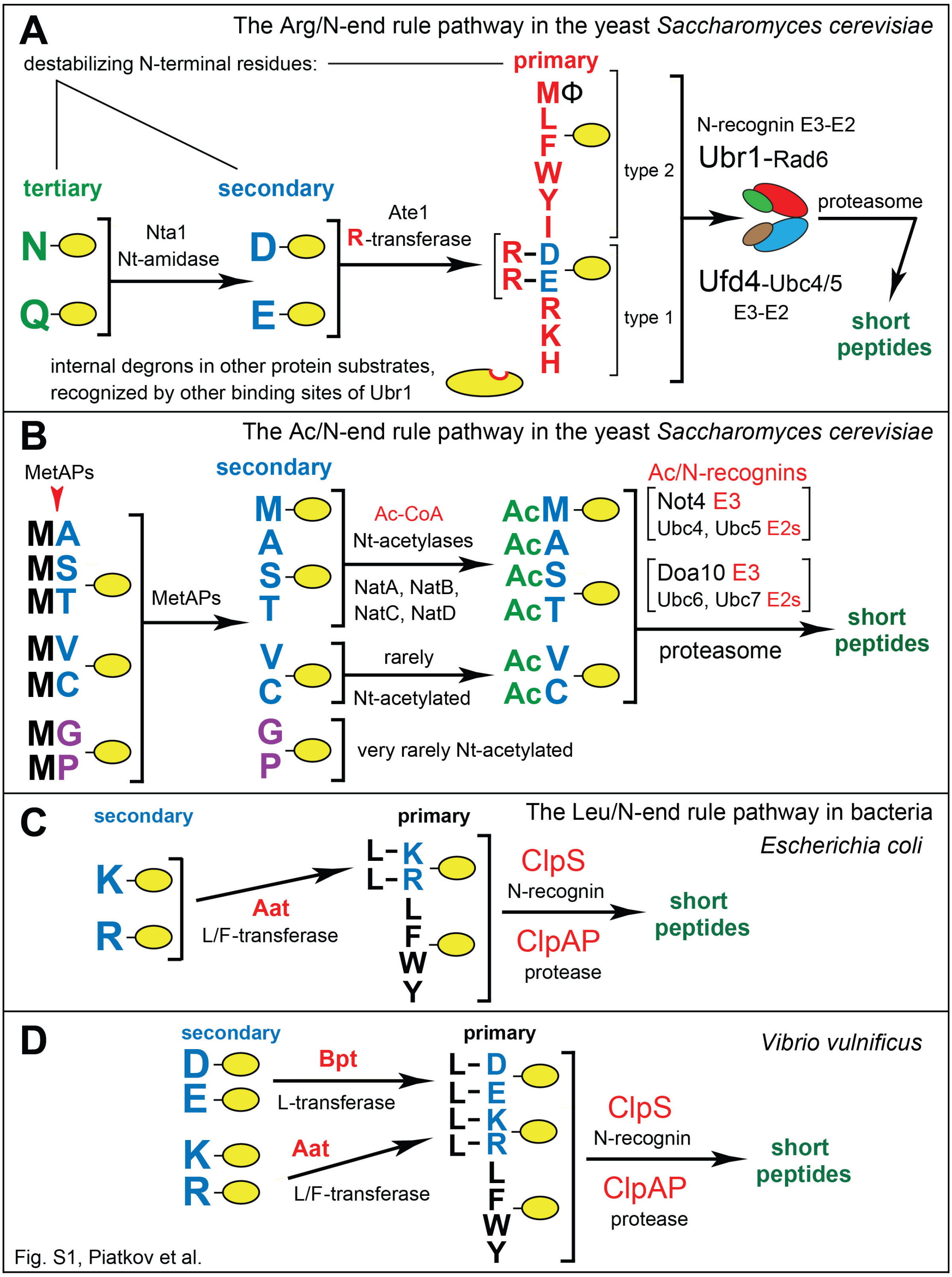

Figure S1. The Arg/N-End Rule Pathway and the Ac/N-End Rule Pathway. N-terminal residues are indicated by single-letter abbreviations. A yellow oval denotes the rest of a protein substrate.

The N-end rule pathway recognizes proteins containing N-terminal degradation signals called N-degrons, polyubiquitylates these proteins and thereby causes their degradation by the 26S proteasome. Recognition components of the N-end rule pathway are called N-recognins. Eukaryotic N-recognins are E3 ubiquitin (Ub) ligases that can target N-degrons. The main determinant of an N-degron is a destabilizing N-terminal residue of a protein. In eukaryotes, the N-end rule pathway consists of two branches, described in panels **A** and **B**.

(A) The Arg/N-end rule pathway in *S. cerevisiae* [1-5]. The prefix "Arg" in the pathway's name refers to Nt-arginylation of N-end rule substrates. The Arg/N-end rule pathway targets specific unacetylated N-terminal residues. It is mediated by the Ubr1 N-recognin, a 225 kDa RING-type E3 Ub ligase and a part of the targeting complex containing the Ubr1-Rad6 and Ufd4-Ubc4/5 holoenzymes. The Ubr1 (N-recognin) component of this complex recognizes (binds to) the "primary" destabilizing N-terminal residues Arg, Lys, His, Leu, Phe, Tyr, Trp and Ile, as well as the unmodified N-terminal Met residue, if Met is followed by a bulky hydrophobic (Φ) residue [5]. The terms "secondary" and "tertiary" refer to the indicated enzymatic modifications of specific N-terminal residues. N-terminal Cys can be arginylated by the Ate1 arginyltransferase (R-transferase) only after the oxidation of Cys to Cys-sulfinate or Cys-sulfonate, in reactions that involve nitric oxide (NO) and oxygen [2-4, 6, 7]. Regulated oxidation of N-terminal Cys takes place in multicellular eukaryotes but not in fungi such as *S. cerevisiae*, which apparently do not produce NO under normal conditions.

(B) The Ac/N-end rule pathway in *S. cerevisiae* [3, 5, 8-10]. This pathway recognizes substrates through their N^{α}-terminally acetylated (Nt-acetylated) residues. The corresponding degradation signals and E3 Ub ligases are called Ac/N-degrons and Ac/N-recognins, respectively. Red arrow on the left indicates the removal of N-terminal Met by Met-aminopeptidases (MetAPs). N-terminal Met is retained if a residue at position 2 is larger than Val [11-13]. The term "secondary" refers to the requirement for a modification (Nt-acetylation) of a destabilizing N-terminal residue before a protein can be recognized by an Ac/N-recognin.

(C) and **(D)** The bacterial Leu/N-end rule pathway, in *Escherichia coli* **(C)** and in *Vibrio vulnificus* **(D)** [14-32]. The Aat L/F-transferase conjugates (largely) Leu to N-terminal Arg or Lys. N-end rule substrates bearing primary (bulky hydrophobic) destabilizing N-terminal residues are recognized by the ClpS N-recognin and are delivered for degradation to the ClpAP protease. In *V. vulnificus*, the Leu/N-end rule pathway contains both the Aat L/F-transferase and the Bpt L-transferase. As a result, N-terminal Asp and Glu, which are not destabilizing residues in *E. coli*, function as secondary destabilizing residues in *V. vulnificus* [16].

Regulated degradation of proteins or their natural fragments by the N-end rule pathway mediates a broad range of biological functions (refs. [2-5, 8-10, 31] and refs therein).

Table S1. Bacterial strains used in this study.

***E. coli* strains**	**Genotype**	**Source or Ref.**
MG1655	*F- λ- rph-1*	[33]
MG-D	*F- λ- rph-1* Δ(def-fmt)::cm *ΔgloB*::frt	Gift from D. Mazel
DH5α	*φ80 lacZΔM15 endA1 recA1 gyrA96 thi-1 hsdR17 (*r_k^- *,* m_k^- *) relA1 supE44 deoR* Δ*(lacZYA–argF)U169*	Promega
CAG12184	$λ^-$ *rph-1 tolC210::Tn10 (tet)*	[34]
KPS73	*λ- rph-1 tolC210::Tn10 (tet) Δfmt ::Km*	This study
KPS74	*λ- rph-1 tolC210::Tn10 (tet) Δdef-Δfmt ::Km*	This study

Table S2. Plasmids used in this study.

Plasmid name	Description	Source or Ref.
pJT184	Cm^R; pACYC184-based plasmid with 2.8-kb BamHI/SalI insert containing *S. cerevisiae* Ubp1.	[14]
pBADET	Ap^R; pBAD2-based plasmid containing arabinose-inducible promoter and expanded multiple cloning site.	Gift from V. Ksenzenko
pACYC177	Ap^R, Km^R; low copy vector containing p15A repl. origin.	[35, 36]
pKD46	Ap^R; ts replication (repA101ts); encodes lambda Red genes (*exo*, *bet*, *gam*); native terminator (tL3) after *exo* gene; arabinose-inducible promoter for expression (P*ara*); encodes *araC* for repression of the Para promoter. This plasmid was used for chromosomal deletions of specific genes.	[37]
pKD3	Ap^R, Cm^R; R6K origin-based plasmid containing *cat*-cassette flanked by FRT sites. This plasmid was used as a template in PCR to create gene-specific *cat*-cassettes.	[37]
pCH178	Ap^R; p314CUP1-based plasmid expressing Ub-CK-e^K-ha-Ura3.	[9]
pKP249	Ap^R; pBADET-based plasmid expressing $P1^{T2}$-e^K-ha-Ura3 from P*ara* promoter.	This study
pKP250	Ap^R; pBADET-based plasmid expressing $P1^{T2D}$-e^K-ha-Ura3 from P*ara* promoter.	This study
pKP251	Ap^R; pBADET-based plasmid expressing Ub-$P1^{T2}$-e^K-ha-Ura3 from P*ara* promoter.	This study
pKP252	Ap^R; pBADET-based plasmid expressing Ub-$P1^{T2D}$-e^K-ha-Ura3 from P*ara* promoter.	This study
pKP257	Ap^R; pACYC177-based plasmid expressing $P1^{T2}$-e^K-ha-Ura3 from P_{KmR} promoter.	This study
pKP258	Ap^R; pACYC177-based plasmid	This study

	expressing P1^{T2D}-e^{K}-ha-Ura3 from P$_{KmR}$ promoter.	
pKP286	Ap^{R}; pBADET-based plasmid expressing PpiB-8his-flag from P*ara* promoter.	This study
pKP287	Ap^{R}; pBADET-based plasmid expressing PpiB^{V2D}-8his-flag from P*ara* promoter.	This study
pKP335	Ap^{R}; pBADET-based plasmid expressing PpiB-8his-flag-Ub-PpiB-8his-flag from P*ara* promoter	This study
pKP458	Ap^{R}; pBADET-based plasmid expressing ^{MVTV}PpiB-8his-flag and ^{MDDD}PpiB-8his-flag-Ub from two identical and independent P*ara* promoters.	This study
pKP459	Ap^{R}; pBADET-based plasmid expressing ^{MVTV}PpiB-8his-flag and ^{MYFY}PpiB-8his-flag-Ub from two identical and independent P*ara* promoters.	This study

Supplemental References

1. Hwang CS, Shemorry A, Varshavsky A (**2010**). The N-end rule pathway is mediated by a complex of the RING-type Ubr1 and HECT-type Ufd4 ubiquitin ligases. **Nat. Cell Biol.** 12: 1177-1185. doi: 10.1038/ncb2121.
2. Tasaki TS, Sriram SM, Park KS, Kwon YT (**2012**). The N-end rule pathway. **Annu. Rev. Biochem.** 81: 261-289. doi: 10.1146/annurev-biochem-051710-093308.
3. Varshavsky A (**2011**). The N-end rule pathway and regulation by proteolysis. **Prot. Sci.** 20: 1298-1345. doi: 10.1002/pro.666.
4. Gibbs DJ, Bacardit J, Bachmair A, Holdsworth MJ (**2014**). The eukaryotic N-end rule pathway: conserved mechanisms and diverse functions. **Trends Cell Biol.** 24: 603-611. doi: 10.1016/j.tcb.2014.05.001.
5. Kim HK, Kim RR, Oh JH, Cho H, Varshavsky A, Hwang CS (**2014**). The N-terminal methionine of cellular proteins as a degradation signal. **Cell** 156: 158-169. doi: 10.1016/j.cell.2013.11.031.
6. Hu R-G, Sheng J, Xin Q, Xu Z, Takahashi TT, Varshavsky A (**2005**). The N-end rule pathway as a nitric oxide sensor controlling the levels of multiple regulators. **Nature** 437: 981-986. PMID: 16222293.
7. Lee MJ, Tasaki T, Moroi K, An JY, Kimura S, Davydov IV, Kwon YT (**2005**). RGS4 and RGS5 are in vivo substrates of the N-end rule pathway. **Proc. Natl. Acad. Sci. USA** 102: 15030-15035. PMID: 16217033.
8. Shemorry A, Hwang CS, Varshavsky A (**2013**). Control of protein quality and stoichiometries by N-terminal acetylation and the N-end rule pathway. **Mol. Cell** 50: 540-551. doi: 10.1016/j.molcel.2013.03.018.
9. Hwang CS, Shemorry A, Varshavsky A (**2010**). N-terminal acetylation of cellular proteins creates specific degradation signals. **Science** 327: 973-977. doi: 10.1126/science.1183147.
10. Park SE, Kim JM, Seok OH, Cho H, Wadas B, Kim SY, Varshavsky A, Hwang CS (**2015**). Control of mammalian G protein signaling by N-terminal acetylation and the N-end rule pathway. **Science** 347: 1249-1252. doi: 10.1126/science.aaa3844.
11. Chen S, Vetro JA, Chang Y-H (**2002**). The specificity in vivo of two distinct methionine aminopeptidases in Saccharomyces cerevisiae. **Arch. Biochem. Biophys.** 398: 87-93. PMID: 11811952.
12. Xiao Q, Zhang F, Nacev BA, Liu JO, Pei D (**2010**). Protein N-terminal processing: substrate specificity of Escherichia coli and human methionine aminopeptidases. **Biochemistry** 49: 5588-5599. doi: 10.1021/bi1005464.
13. Frottin F, Martinez A, Peynot P, Mitra S, Holz RC, Giglione C, Meinnel T (**2006**). The proteomics of N-terminal methionine cleavage. **Mol. Cell. Proteomics** 5: 2336-2349. PMID: 16963780.
14. Tobias JW, Shrader TE, Rocap G, Varshavsky A (**1991**). The N-end rule in bacteria. **Science** 254: 1374-1377. PMID: 1962196.
15. Shrader TE, Tobias JW, Varshavsky A (**1993**). The N-end rule in *Escherichia coli*: cloning and analysis of the leucyl, phenylalanyl-tRNA-protein transferase gene *aat*. **J. Bact.** 175: 4364-4374. PMID: 8331068.

16. Graciet E, Hu RG, Piatkov K, Rhee JH, Schwarz EM, Varshavsky A (**2006**). Aminoacyl-transferases and the N-end rule pathway of prokaryotic/eukaryotic specificity in a human pathogen. **Proc. Natl. Acad. Sci. USA** 103: 3078-3083. PMID: 16492767.
17. Erbse A, Schmidt R, Bornemann T, Schneider-Mergener J, Mogk A, Zahn R, Dougan DA, Bukau B (**2006**). ClpS is an essential component of the N-end rule pathway in *Escherichia coli*. **Nature** 439: 753-756. PMID: 16467841.
18. Suto K, Shimizu Y, Watanabe K, Ueda T, Fukai S, Nureki O, Tomita K (**2006**). Crystal structures of leucyl/phenylalanyl-tRNA-protein transferase and its complex with an aminoacyl-tRNA analog. **EMBO J.** 25: 5942-5950. PMID: 17110926.
19. Dong XK-M, M., Muramatsu T, Mori H, Shirouzu M, Bessho Y, Yokoyama S (**2007**). The crystal structure of leucyl/phenylalanyl-tRNA-protein transferase from Escherichia coli **Prot. Sci.** 16: 528-534. PMID: 17242373.
20. Wang KH, Roman-Hernandez G, Grant RA, Sauer TT, Baker TA (**2008**). The molecular basis of N-end rule recognition. **Mol. Cell** 32: 406-414. doi: 10.1016/j.molcel.2008.08.032.
21. Wang KH, Oakes ESC, Sauer RT, Baker TA (**2008**). Tuning the strength of a bacterial N-end rule signal. **J. Biol. Chem.** 283: 24600-24607. doi: 10.1074/jbc.M802213200.
22. Hou JY, Sauer RT, Baker TA (**2008**). Distinct structural elements of the adaptor ClpS are required for regulating degradation by ClpAP. **Nat. Struct. Mol. Biol.** 15: 288-294. doi: 10.1038/nsmb.1392.
23. Schmidt R, Zahn R, Bukau B, Mogk A (**2009**). ClpS is the recognition component for Escherichia coli substrates of the N-end rule degradation pathway. **Mol. Microbiol.** 72: 506-517. doi: 10.1111/j.1365-2958.2009.06666.x.
24. Román-Hernández G, Grant RA, Sauer RT, Baker TA (**2009**). Molecular basis of substrate selection by the N-end rule adaptor protein ClpS. **Proc. Natl. Acad. Sci. USA** 106: 8888-8893. doi: 10.1073/pnas.0903614106.
25. Schuenemann VJ, Kralik SM, Albrecht R, Spall SK, Truscott KN, Dougan DA, Zeth K (**2009**). Structural basis of N-end rule substrate recognition in Escherichia coli by the ClpAP adaptor protein ClpS. **EMBO Rep.** 10: 508-514. doi: 10.1038/embor.2009.62.
26. Ninnis RL, Spall SK, Talbo GH, Truscott KN, Dougan DA (**2009**). Modification of PATase by L/F-transferase generates a ClpS-dependent N-end rule substrate in Escherichia coli. **EMBO J.** 28: 1732-1744. doi: 10.1038/emboj.2009.134.
27. Román-Hernández G, Hou JY, Grant RA, Sauer RT, Baker TA (**2011**). The ClpS adaptor mediates staged delivery of N-end rule substrates to the AAA+ ClpAP protease. **Mol. Cell** 43: 217-228. doi: 10.1016/j.molcel.2011.06.009.
28. Humbard MA, Surkov S, De Donatis GM, Jenkins L, Maurizi MR (**2013**). The N-degradome of Escherichia coli: limited proteolysis in vivo generates a large pool of proteins bearing N-degrons. **J. Biol. Chem.** 288: 28913-28924. doi: 10.1074/jbc.M113.492108.
29. Rivera-Rivera I, Román-Hernández G, Sauer RT, Baker TA (**2014**). Remodeling of a delivery complex allows ClpS-mediated degradation of N-degron substrates. **Proc. Natl. Acad. Sci. USA** 111: E3853-E3859. doi: 10.1073/pnas.1414933111.
30. Fung AW, Fahlman RP (**2015**). The molecular basis for the post-translational addition of amino acids by L/F-transferase in the N-end rule pathway. **Curr. Prot. Pept. Sci.** 16: 163-180. PMID: 25579118.

31. Dougan DA, Micevski D, Truscott KN (**2011**). The N-end rule pathway: from recognition by N-recognins to destruction by AAA+ proteases. **Biochim. Biophys. Acta** 1823: 83-91. doi: 10.1016/j.bbamcr.2011.07.002.
32. Mogk A, Schmidt R, Bukau B (**2007**). The N-end rule pathway of regulated proteolysis: prokaryotic and eukaryotic strategies. **Trends Cell Biol.** 17: 165-172. PMID: 17306546.
33. Blattner FR, Plunkett G, 3rd, Bloch CA, Perna NT, Burland V, Riley M, Collado-Vides J, Glasner JD, Rode CK, Mayhew GF, Gregor J, Davis NW, Kirkpatrick HA, Goeden MA, Rose DJ, Mau B, Shao Y (**1997**). The complete genome sequence of Escherichia coli K-12. **Science** 277: 1453-1462. PMID: 9278503.
34. Singer M, Baker TA, Schnitzler G, Deischel SM, Goel M, Dove W, Jaacks KJ, Grossman AD, Erickson JW, Gross CA (**1989**). A collection of strains containing genetically linked alternating antibiotic resistance elements for genetic mapping of Escherichia coli. **Microbiol. Rev.** 53: 1-24. PMID: 2540407.
35. Chang AC, Cohen SN (**1978**). Construction and characterization of amplifiable multicopy DNA cloning vehicles derived from the P15A cryptic miniplasmid. **J. Bacteriol.** 134: 1141-1156. PMID: 149110.
36. Rose RE (**1988**). The nucleotide sequence of pACYC184. **Nucl. Acids Res.** 16: 355.
37. Datsenko KA, Wanner BL (**2000**). One-step inactivation of chromosomal genes in *Escherichia coli* K-12 using PCR products. **Proc. Natl. Acad Sci. USA** 97: 6640-6645. PMID: 10829079.
